# Gut Microbiota, Diet and Lipid Metabolism in Adolescents with NAFLD and Their Role in Preventive Strategies

**DOI:** 10.3390/ijms27083511

**Published:** 2026-04-14

**Authors:** Natalia Kurhaluk, Zbigniew Mazur, Renata Kołodziejska, Halina Tkaczenko

**Affiliations:** 1 Institute of Biology, Pomeranian University in Słupsk, Arciszewski St. 22B, 76-200 Słupsk, Poland; zbigniewmazur1@wp.pl; 2 Department of Medical Biology and Biochemistry, Collegium Medicum in Bydgoszcz, Nicolaus Copernicus University in Toruń, 85-092 Bydgoszcz, Poland; renatak@cm.umk.pl

**Keywords:** NAFLD, MASLD, prebiotics, postbiotics, epigenetics, metabolomics, microbial metabolites

## Abstract

Adolescence is a metabolically vulnerable period, during which rapid physiological maturation coincides with the dynamic remodelling of the gut microbiome. This narrative review summarises evidence from 2015 to 2025 to clarify how disturbances to the gut–liver axis driven by dysbiosis contribute to the development and progression of non-alcoholic fatty liver disease (NAFLD) in young people. Based on a systematic search of the databases PubMed, Scopus and Web of Science, we outline the basis of bidirectional communication between the gut and liver and emphasise how microbial imbalance alters the handling of lipids in the liver by enhancing de novo lipogenesis, impairing fatty acid oxidation and disrupting AMPK signalling and mitochondrial function. Consistent findings from clinical and experimental studies show that adolescents with NAFLD exhibit reduced microbial diversity, the enrichment of ethanol- and LPS-producing taxa, and altered short-chain fatty acid profiles. Each of these is associated with hepatic inflammation and metabolic reprogramming. Microbial molecules, including LPS, secondary bile acids and branched-chain amino acid metabolites, activate TLR4–NF-κB pathways, promote Kupffer cell activation and intensify oxidative stress. These mechanisms intersect with factors specific to adolescence, such as increased adiposity, hormonal shifts and diet-induced metabolic strain. Dietary patterns emerge as key modulators of these processes. Westernised diets promote dysbiosis and endotoxemia, whereas Mediterranean, fibre-rich and plant-based diets enhance SCFA production, strengthen epithelial integrity and modulate adiponectin-dependent hepatic metabolism. Micronutrient-sensitive epigenetic regulation, particularly that involving folate, choline and polyphenols, also plays a role in shaping lipid homeostasis and inflammatory tone. We also highlight emerging evidence that the activation of cytoprotective pathways, especially Nrf2, is dependent on lifestyle factors and links antioxidant-rich functional foods and physical activity to improved mitochondrial resilience and microbiome stability. We evaluate therapies targeting the microbiome, including probiotics, prebiotics, synbiotics and postbiotics, which reduce endotoxemia, restore microbial balance and complement dietary strategies. Thus, these findings emphasise the importance of age-specific, mechanistically informed interventions that integrate diet quality, microbial ecology, and the molecular pathways that govern metabolic health in adolescents with NAFLD.

## 1. Introduction

Non-alcoholic fatty liver disease (NAFLD) is defined as the pathological accumulation of lipids in more than 5% of hepatocytes in individuals who consume moderate or less alcohol and for whom liver injury cannot be attributed to viral infections, hepatotoxic medications or autoimmune disorders [1,2]. Over the past decade, NAFLD has become one of the fastest-increasing chronic liver conditions among adolescents in high-income countries. While early studies by Anderson et al. [3] emphasised the dominant role of obesity, more recent analyses by Luo et al. [4] highlight a broader constellation of risk factors, including sedentary behaviour, a high intake of sugar-sweetened beverages, and widespread exposure to ultra-processed foods. This shift in perspective illustrates how the epidemiology of NAFLD in adolescents has evolved alongside profound changes in lifestyle and food environments.

Epidemiological data emphasise the urgency of the problem because NAFLD affects around 10% of people under 18, with prevalence increasing sharply from under 1% in children aged 2–4 to almost 17% in adolescents [5,6]. Diagnostic heterogeneity further complicates matters: ultrasonography-based studies report a prevalence of as little as 1.8%, whereas in obese or bariatric cohorts, rates can reach 60–80% [7,8]. According to the latest metabolic dysfunction-associated steatotic liver disease (MASLD) classification, MASLD is now the most common liver disorder globally, affecting 38% of adults and 13% of children and adolescents [9]. These discrepancies highlight the complexity of diagnosing NAFLD in young people and the need for harmonised diagnostic criteria.

In 2023, an international consensus group proposed replacing the long-standing term ‘non-alcoholic fatty liver disease’ (NAFLD) with ‘metabolic dysfunction-associated steatotic liver disease’ (MASLD), reflecting a shift towards a definition that is more oriented towards pathophysiology [9]. As our review covers literature published over the past decade, most of the original studies still use the term ‘NAFLD’. For this reason, we use MASLD as the primary term in the present manuscript, while retaining NAFLD when referring to historical definitions or citing studies that use this nomenclature. This approach ensures conceptual clarity and fidelity to the terminology used in the original research.

The pathogenesis of NAFLD is multifactorial, involving complex interactions between environmental factors—particularly diet and gut microbiota—and host metabolic and genetic predispositions [10]. Adolescence represents a particularly vulnerable developmental stage. Kemp et al. [11] note that this period is characterised by significant hormonal and metabolic changes, whereas Neufeld et al. [12] emphasise increasing autonomy in dietary choices and heightened susceptibility to social and environmental influences. Together, these factors indicate that adolescence is a critical window during which long-term metabolic trajectories are established.

Dietary patterns among adolescents are consistently suboptimal. Studies by Gu and Tucker [13] and Kim et al. [14] have documented low fruit and vegetable intake, as well as poor adherence to Mediterranean dietary principles. In contrast, Winpenny et al. [15] highlight the increasing consumption of ultra-processed foods that are high in saturated fats and simple sugars. Irregular eating habits, such as skipping breakfast, frequent consumption of fast food, and restrictive dieting, further exacerbate insulin resistance, visceral adiposity, and chronic low-grade inflammation [16,17]. Excessive intake of saturated fats, cholesterol and refined sugars, combined with micronutrient deficiencies, can lead to dyslipidaemia, oxidative stress and hepatocellular injury [18,19]. Furthermore, Russo et al. [20] and Melton et al. [21] have demonstrated that unhealthy dietary habits during adolescence can lead to epigenetic modifications that persist into adulthood, thereby increasing the risk of metabolic disorders. Even modest weight gain of 3–5 kg significantly increases the risk of NAFLD, regardless of baseline body mass index (BMI) [22].

There is growing evidence that gut microbiota dysbiosis and disruption to the gut–liver axis play a central role in the development of NAFLD in young people. Tokuhara [23] highlights that increased intestinal permeability and endotoxin lipopolysaccharide (LPS) translocation trigger hepatic inflammation. Meanwhile, Hrncir et al. [10] and Fan and Pedersen [24] demonstrate that healthy microbiota support lipid metabolism, energy homeostasis and intestinal barrier integrity. Dysbiosis promotes hepatic lipid accumulation, insulin resistance, oxidative stress and inflammation—mechanisms that, when combined with unhealthy dietary patterns, create a self-perpetuating cycle that accelerates the progression from simple steatosis to non-alcoholic steatohepatitis (NASH) and further metabolic complications [5,25].

Thus, ongoing investigation into NAFLD remains vital for several reasons. The increasing prevalence among young people indicates a concerning trend of earlier onset of metabolic dysfunction, implying that current adolescents may be at risk of lifelong cardiometabolic issues. At the same time, the absence of any approved pharmacotherapy for NAFLD or its progressive form, NASH, highlights the urgent need for mechanistic research to reveal actionable therapeutic targets. The disease’s complex pathogenesis, shaped by metabolic, inflammatory, immunological and microbial interactions, further reinforces the importance of continued study. A deeper understanding of these interconnected pathways will enable the development of effective, personalised interventions. Therefore, the growing public health impact of NAFLD, which is projected to become the leading indication for liver transplantation, signals an impending burden on healthcare systems worldwide, strengthening the need for sustained scientific attention.

The novelty of this narrative review lies in its explicit focus on the unique molecular and physiological mechanisms that make adolescence a critical window for the development and progression of NAFLD. Integrating evidence on diet-induced metabolic dysregulation, microbiota-driven modulation of bile acids and inflammatory signalling, and the hormonal and epigenetic shifts that characterise puberty, the review reveals how these processes converge to accelerate hepatic steatosis specifically in young people. This adolescent-centred, mechanistic perspective is rarely addressed in current NAFLD literature, which predominantly focuses on adults. Therefore, this review provides new insight into early pathogenic drivers and opportunities for targeted prevention.

This narrative review aims to synthesise and critically evaluate the current evidence regarding the interaction between diet, gut microbiota and lipid metabolism in adolescents with NAFLD, with a particular focus on the molecular pathways linking these factors. Integrating nutritional, microbiological, and metabolic perspectives, the review will elucidate mechanistic connections, such as microbiota-driven modulation of bile acid signalling (farnesoid X receptor—FXR, Takeda G-protein-coupled receptor 5—TGR5), short-chain fatty acid–mediated regulation of hepatic lipogenesis, dietary alterations to insulin and mTOR pathways, and inflammatory activation through endotoxin-TLR4 signalling. This multidimensional approach will enable the identification of converging biological pathways, the highlighting of inconsistencies and gaps in existing knowledge, and the outlining of opportunities for early preventive interventions targeting modifiable behaviours and microbial ecology.

## 2. Search Strategy and Study Selection

The aim of this narrative review was to identify recent and relevant evidence on the relationships between gut microbiota, dietary patterns, physical activity, and metabolic biomarkers in adolescents with non-alcoholic fatty liver disease (NAFLD). Literature searches were conducted in PubMed, Scopus, Web of Science and Google Scholar, covering peer-reviewed publications from 2014 to 2025. Combinations of keywords relating to “adolescents”, “NAFLD/MASLD”, “gut microbiota”, “dietary patterns”, “lipid metabolism”, “physical activity” and “hormonal signalling” were used. The titles and abstracts of the identified studies were screened to select those involving adolescent or mixed pediatric populations that provided data relevant to the diet–microbiota–metabolism axis in NAFLD. Full texts were reviewed when eligibility was uncertain.

Studies examining adolescents or pediatric cohorts and contributing mechanistic, clinical or metabolic insights into NAFLD were included. Articles focusing exclusively on adults, unrelated liver diseases, case reports, conference abstracts, or studies lacking clinical or mechanistic relevance were excluded. This approach enabled the integration of clinical, experimental and developmental findings, providing a comprehensive synthesis of current knowledge on NAFLD risk in young people.

Google Scholar was only used as a supplementary source to ensure that no major peer-reviewed studies were overlooked. Records retrieved after 2025 were excluded because Google Scholar frequently indexes uncatalogued materials, duplicates, and non-peer-reviewed content that fall outside the scope of this review. The time frame of 2014–2025 reflects the period during which the majority of studies focusing on NAFLD and the microbiome in adolescents have emerged.

## 3. Gut–Liver Axis and Microbiome-Driven Mechanisms

### 3.1. Bidirectional Gut–Liver Communication

**Figure 1:**
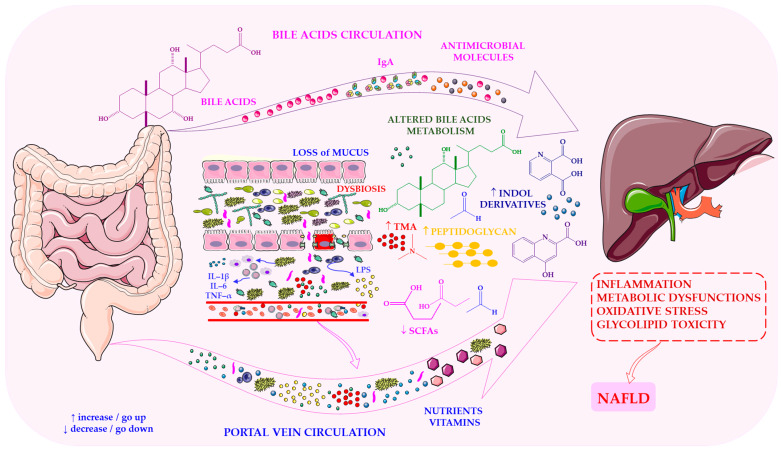
The gut–liver axis in NAFLD: dysbiosis-driven inflammation and metabolic dysfunction. The gut–liver axis is a dynamic, interdependent system in which dysbiosis disrupts epithelial barrier integrity, enhances endotoxin translocation, and alters bile acid and short-chain fatty acid (SCFA) signaling. These disturbances initiate a self-perpetuating cycle of inflammation and metabolic dysfunction, thereby increasing susceptibility to non-alcoholic fatty liver disease (NAFLD). Image provided by Servier Medical Art (https://smart.servier.com/), licensed under CC BY 4.0 (https://creativecommons.org/licenses/by/4.0/, accessed on 10 January 2026). Abbreviations: IgA–immunoglobulin A; IL-1β–interleukin-1 beta; IL-6–interleukin-6; LPS–lipopolysaccharide; NAFLD–non-alcoholic fatty liver disease; SCFAs–short-chain fatty acids; TMA–trimethylamine; TNF-α–tumor necrosis factor alpha.

The gut–liver axis forms a tightly integrated metabolic and immunological network in which intestinal microbiota and hepatic function continuously influence one another. McDonald et al. [26] emphasise that the liver acts as a ‘microbial firewall’, clearing bacteria and microbial products that arrive via the portal vein, primarily through Kupffer cell phagocytosis. Furthermore, Balmer et al. [27] note that this communication depends on the enterohepatic circulation of bile acids and metabolites, which shape the intestinal environment and regulate microbial composition. Under physiological conditions, primary bile acids are reabsorbed in the small intestine, while residual fractions are converted by commensal bacteria into secondary bile acids that re-enter the liver. The gut-liver axis in NAFLD, including dysbiosis-driven inflammation and metabolic dysfunction, is illustrated in Figure 1.

It was highlighted by Jones & Neish [28] that any disruption of this bidirectional communication-whether due to intestinal or hepatic pathology-leads to dysbiosis and reciprocal metabolic dysfunction. Dysbiosis alters the production of microbial metabolites, such as short-chain fatty acids (SCFAs), acetaldehyde, and secondary bile acids. Once delivered to the liver, these metabolites exacerbate inflammation and metabolic stress. Amrousy et al. [5] report that diet, genetics, and exposure to xenobiotics can weaken intestinal tight junctions, thereby increasing permeability and facilitating the translocation of bacteria and their products.

A broad spectrum of bioactive microbial molecules, including LPS, peptidoglycan, extracellular vesicles, indole derivatives, trimethylamine (TMA), carotenoids, phenolic compounds and modified bile acids, are described by Ji et al. [29] as reaching the liver and modulating immune activation, lipid metabolism and oxidative stress. Ji et al. [29] also emphasise that secondary bile acids and microbial aldehydes can directly impair hepatocyte function, thereby reinforcing the pathological loop between the gut and the liver.

Table 1 provides a comprehensive overview of systematic summaries of experimental and clinical investigations linking gut microbiota alterations to lipid metabolism in youth with NAFLD (2015–2025).

Thus, the gut–liver axis is a dynamic, interdependent system in which dysbiosis disrupts epithelial integrity, increases endotoxin translocation and alters bile acid and SCFA signalling. These disturbances then initiate a self-reinforcing cycle of inflammation and metabolic dysfunction that increases the likelihood of NAFLD developing. Collectively, the evidence indicates that gut microbiota is essential for maintaining gut–liver axis homeostasis, and its disruption triggers increased intestinal permeability, endotoxin translocation, oxidative stress and hepatic inflammation; in parallel, liver dysfunction further reshapes microbial composition, establishing a self-perpetuating pathogenic loop that drives the onset of NAFLD and accelerates its progression toward NASH by amplifying hepatic inflammation, intensifying oxidative and endoplasmic reticulum stress, promoting hepatocyte ballooning and apoptosis, and driving stellate-cell activation and fibrogenesis, ultimately shifting NAFLD from a metabolically driven steatotic state to an inflammatory and fibrotic phenotype.

### 3.2. Mechanisms of Dysbiosis-Driven Hepatic Injury

**Figure 2:**
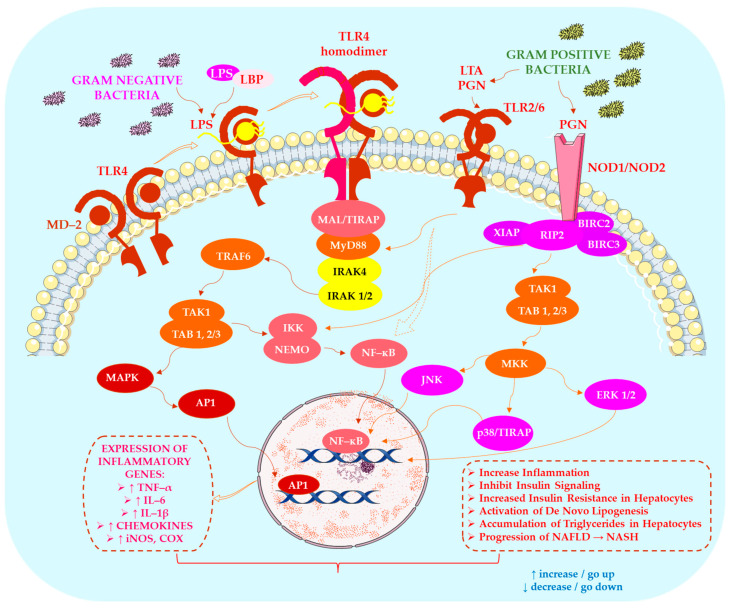
Microbial-derived molecules as drivers of hepatic inflammation and metabolic reprogramming. Microbial molecules originating from gut dysbiosis activate innate immune signaling in the liver, promoting chronic inflammation and metabolic reprogramming that drive NAFLD progression. Lipopolysaccharide (LPS), a component of the outer membrane of Gram-negative bacteria, enhances pro-inflammatory mediators by activating TLR4 and the NF-κB pathway. In parallel, lipoteichoic acid (LTA) and peptidoglycan (PGN) derived from Gram-positive bacteria signal through the TLR2–MyD88 and NOD1/NOD2 pathways, further amplifying hepatic inflammation, insulin resistance, and lipid accumulation. Image provided by Servier Medical Art (https://smart.servier.com/), licensed under CC BY 4.0 (https://creativecommons.org/licenses/by/4.0/, accessed on 10 January 2026). Abbreviations: AP-1–activator protein 1; BIRC2–baculoviral IAP repeat containing protein 2 (cIAP1); BIRC3–baculoviral IAP repeat containing protein 3 (cIAP2); COX–cyclooxygenase; ERK1/2–extracellular signal-regulated kinases 1 and 2; IKK–IκB kinase; IL-1β–interleukin-1 beta; IL-6–interleukin-6; iNOS–inducible nitric oxide synthase; IRAK1/2–interleukin-1 receptor-associated Kinases 1 and 2; IRAK4–interleukin-1 receptor-associated kinase 4; JNK–c-Jun N-terminal kinase; LBP–lipopolysaccharide-binding protein; LPS–lipopolysaccharide; LTA–lipoteichoic acid; MAL–myD88-adaptor-like protein (TIRAP); MAPK–mitogen-activated protein kinase; MD-2–myeloid differentiation factor 2; MKK–MAPK kinase; MyD88–myeloid differentiation primary response 88; NAFLD–non-alcoholic fatty liver disease; NASH–non-alcoholic steatohepatitis; NEMO–NF-κB essential modulator (IKKγ); NF-κB–nuclear factor kappa-light-chain-enhancer of activated B cells; NOD1–nucleotide-binding oligomerization domain-containing protein 1; NOD2–nucleotide-binding oligomerization domain-containing protein 2; p38–p38 mitogen-activated protein kinase; PGN–peptidoglycan; RIP2–receptor-interacting protein kinase 2; TAB1–TAK1-binding protein 1; TAB2/3–TAK1-binding proteins 2 and 3; TAK1–transforming growth factor-β-activated kinase 1; TLR2/6–toll-like receptors 2 and 6; TLR4–toll-like receptor 4; TNF-α–tumor necrosis factor alpha; TRAF6–TNF receptor-associated factor 6; TIRAP–toll/Interleukin-1 Receptor (TIR) domain-containing adaptor protein; XIAP–x-linked inhibitor of apoptosis protein.

Numerous studies have demonstrated that LPS, a major component of the outer membrane of Gram-negative bacteria, is one of the most potent microbial mediators driving the progression of NAFLD. Namely, studies of authors Ji et al. [29] show that LPS activates Toll-like receptor 4 (TLR4) in hepatocytes, Kupffer cells, and hepatic stellate cells, thereby inducing the production of key pro-inflammatory cytokines, including interleukin-6 (IL-6), interleukin-1 beta (IL-1β), and tumour necrosis factor alpha (TNF-α). Evidence from Sharifnia et al. [38] and Fukunishi et al. [39] indicates that chronic activation of the TLR4 pathway promotes the transition from simple steatosis to NASH. Furthermore, Ceccarelli et al. [40] report that LPS enhances chemokine-mediated recruitment of immune cells, thereby amplifying hepatocellular injury. As shown in Figure 2, microbial-derived molecules contribute to hepatic inflammation and metabolic reprogramming, playing a central role in NAFLD pathogenesis.

In addition to receptor-mediated signalling, circulating LPS-binding protein (LBP) plays a central role in endotoxin transport. Pang et al. [41] describe LBP as a key shuttle molecule whose plasma concentrations correlate with insulin resistance and dyslipidaemia. Findings by Wong et al. [42] and Jin et al. [43] further confirm that elevated LBP levels are indicative of metabolic inflammation. Chenevier-Gobeaux et al. [44] identified soluble CD14 (sCD14, also known as presepsin) as a biomarker reflecting NASH severity, underscoring the clinical relevance of the LPS–TLR4 axis in metabolic liver disease.

LPS is also well known to disrupt intestinal tight junctions. Studies of team Nighot et al. [45] demonstrated that this effect is mediated through CD14, myosin light chain kinase (MLCK), and interleukin-1 receptor-associated kinase 4 (IRAK-4) signalling, which collectively increase epithelial permeability and facilitate endotoxin translocation. Additional evidence from Guo et al. [46] confirms that LPS-induced MLCK activation further weakens epithelial integrity. Beyond the intestine, LPS entering the portal circulation activates hepatic immune and stromal cells. Wan et al. [47] showed that portal LPS stimulates Kupffer cells and hepatic stellate cells, thereby promoting inflammatory responses and fibrosis. Ji et al. [29] expanded this perspective by demonstrating that components of Gram-positive bacteria also possess strong inflammatory potential: peptidoglycan activates Toll-like receptors 2 and 6 (TLR2 and TLR6) as well as nucleotide-binding oligomerisation domain-containing proteins 1 and 2 (NOD1 and NOD2). Meanwhile, lipoteichoic acid (LTA) enhances hepatic triglyceride lipolysis and contributes to hyperlipidaemia. According to Ji et al. [29], LTA-driven lipid mobilisation further exacerbates metabolic stress in NAFLD.

Studies by Devlin et al. [48] and Beaumont et al. [49] show that indole and its derivatives exhibit notable protective properties, strengthening epithelial tight junctions, reducing inflammation, and improving glucose homeostasis. Their metabolic benefits also extend to hormonal regulation: Whitfield-Cargile et al. [50] and Chimerel et al. [51] observed that indole stimulates glucagon-like peptide-1 (GLP-1) secretion. The immunomodulatory effects of indole-3-aldehyde are attributed to aryl hydrocarbon receptor (AhR) activation, which leads to interleukin-22 (IL-22) and interleukin-10 receptor subunit 1 (IL-10R1) induction [52]. Meanwhile, indole-3-acetic acid suppresses pro-inflammatory cytokines and lipogenesis, as demonstrated by Natividad et al. [53] and Krishnan et al. [54]. Barrier-enhancing and glucose-modulating effects have also been documented for indole-3-propionic acid, with Venkatesh et al. [55] and Abildgaard et al. [56] demonstrating its capacity to reinforce epithelial integrity and support metabolic health.

**Figure 3:**
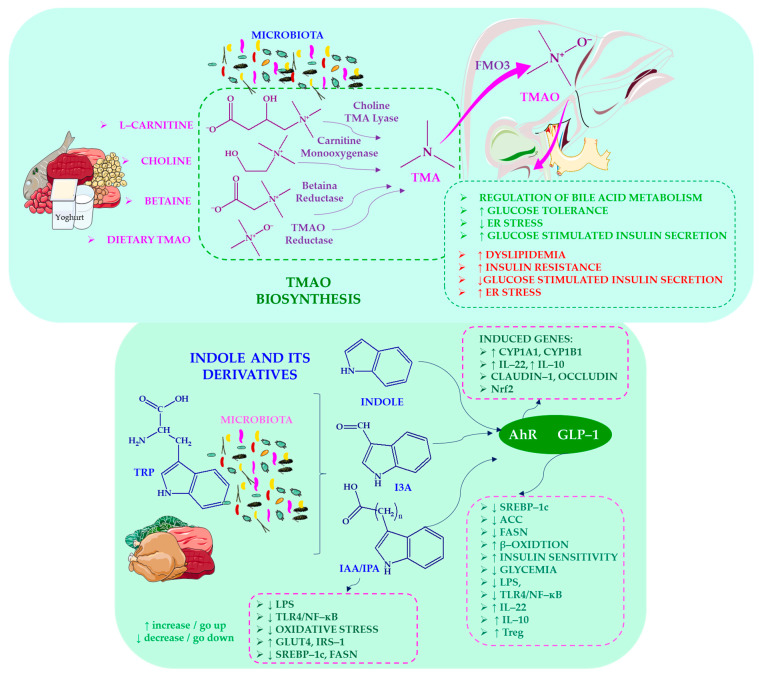
Microbiota-derived tryptophan metabolites and TMAO in the regulation of hepatic inflammation and metabolic homeostasis. Microbiota-derived tryptophan metabolites, including indole and its derivatives, act as ligands for the aryl hydrocarbon receptor (AhR), inducing transcriptional programs that regulate immune responses, epithelial barrier integrity, and inflammation. AhR signaling improves metabolic homeostasis by enhancing insulin sensitivity, activating AMPK, modulating GLP-1 secretion, reducing oxidative stress, and suppressing hepatic lipogenic gene expression. In parallel, trimethylamine (TMA), produced from choline, betaine, and carnitine, is converted in the liver into trimethylamine N-oxide (TMAO), which modulates bile acid metabolism and hepatic triglyceride accumulation, affects glucose tolerance and glucose homeostasis, and contributes to the induction of endoplasmic reticulum (ER) stress. However, under conditions of metabolic stress, TMAO may also exert detrimental effects by promoting insulin resistance, dyslipidemia, and hepatic inflammation. Image provided by Servier Medical Art (https://smart.servier.com/), licensed under CC BY 4.0 (https://creativecommons.org/licenses/by/4.0/, accessed on 10 January 2026). Abbreviations: ACC–acetyl-CoA carboxylase; CYP1A1–cytochrome P450 family 1 subfamily A member 1; CYP1B1–cytochrome P450 family 1 subfamily B member 1; ER–endoplasmic reticulum; FASN–fatty acid synthase; FMO3–flavin-containing monooxygenase 3; GLP-1–glucagon-like peptide-1; GLUT4–glucose transporter type 4; I3A–indole-3-aldehyde; IAA–indole-3-acetic acid; IL-10–interleukin-10; IL-22–interleukin-22; IRS-1–insulin receptor substrate-1 (inhibitory phosphorylation); IPA–indole-3-propionic acid; LPS–lipopolysaccharide; NF-κB–nuclear factor kappa B; Nrf2–nuclear factor erythroid 2–related factor 2; SREBP-1c–sterol regulatory element-binding protein-1c; TLR4–toll-like receptor 4; TMA–trimethylamine; TMAO–trimethylamine N-oxide; TRP–tryptophan; Treg–regulatory T cells.

The scientific team of Fennema et al. [57] describe how TMA, produced from dietary choline, betaine, and carnitine, is converted to trimethylamine N-oxide (TMAO) by hepatic flavin monooxygenase (FMO) enzymes. Barrea et al. [58] emphasise that TMA production is strongly influenced by dietary patterns commonly observed in obesity. Zhu et al. [59] demonstrate that TMAO regulates bile acid metabolism via cholesterol 7α-hydroxylase (CYP7A1) and hepatic transporters. Ji et al. [29] add that choline deficiency impairs very-low-density lipoprotein (VLDL) secretion, thereby promoting triglyceride accumulation. Meanwhile, Warrier et al. [60] and Shih et al. [61] demonstrate that TMAO promotes insulin resistance, dyslipidaemia, and hepatic inflammation. Conversely, Oellgaard et al. [62] link TMAO to altered cholesterol homeostasis. The way in which SCFA and microbiota-driven bile acid modifications modulate FXR and TGR5 signalling, shaping lipid and glucose metabolism, was emphasised by Zhang et al. [63]. As shown in Figure 3, microbiota-derived tryptophan metabolites and TMAO play key roles in regulating hepatic inflammation and maintaining metabolic homeostasis.

Thus, microbial molecules exert both harmful (LPS, peptidoglycan, LTA, TMAO) and protective (indole derivatives, SCFA) effects on hepatic metabolism. Consequently, NAFLD progression reflects an imbalance between pro-inflammatory microbial signals and homeostatic metabolites, ultimately leading to chronic inflammation, lipid dysregulation, and fibrosis.

### 3.3. Microbial Metabolites and Inflammatory Pathways

The rising prevalence of NAFLD is closely linked to dietary shifts toward higher energy intake, particularly from refined carbohydrates, fats and fructose. The increased use of high-fructose corn syrup and artificial sweeteners such as sucralose may further contribute to disease development [64].

Hepatic triglyceride (TG) accumulation in NAFLD arises from multiple sources: 60% of TG originate from white adipose tissue (WAT), 26% from de novo lipogenesis (DNL) and 15% from dietary fat [65,66,67]. Insulin normally suppresses lipolysis and promotes TG storage in adipose tissue, but in insulin resistance (IR) this effect is impaired, leading to increased release of free fatty acids (FFA) from WAT and their subsequent hepatic deposition [67]. DNL is regulated by sterol regulatory element-binding protein-1c (SREBP-1c) and carbohydrate-responsive element-binding protein, which activate acetyl-CoA carboxylase and fatty acid synthase (FAS), thereby enhancing lipid synthesis [67,68,69,70]. Consequently, high-fat diets (HFD) promote obesity, IR and moderate hepatic steatosis, whereas fructose exerts additional detrimental effects by upregulating genes involved in fibrosis, inflammation, endoplasmic reticulum (ER) stress and adipocyte apoptosis [67].

Because fructose is metabolised almost exclusively in the liver, it activates c-Jun N-terminal kinase (JNK) and exacerbates insulin resistance (IR), driving excessive lipogenesis, impaired fatty acid oxidation (FAO), hepatic inflammation and fibrosis [67,71]. Romero-Gómez et al. [22] further demonstrated that high fructose intake increases intestinal permeability, endotoxaemia, hepatic TNF-α production and lipid peroxidation, collectively accelerating NAFLD progression.

Ultra-processed foods (UPFs), characterised by refined carbohydrates, unhealthy fats, and numerous additives, have been associated with gut dysbiosis, impaired intestinal barrier function, altered gastrointestinal motility, and low-grade inflammation. These disruptions may interfere with gut–brain signalling and contribute to functional gastrointestinal disorders (FGIDs) [16]. Thus, diet-induced dysbiosis, IR and excessive hepatic lipid accumulation form the mechanistic basis of NAFLD. High-fat, high-sugar and fructose-rich diets disrupt metabolic homeostasis, impair intestinal barrier function and promote hepatic inflammation and fibrosis.

The evidence collectively demonstrates that diet is one of the most powerful modulators of the gut microbiome and a key determinant of metabolic health. Plant-based and mediterranean dietary patterns have been shown to consistently increase beneficial bacterial taxa, enhance SCFA production and improve microbial diversity, thereby reducing the risk of metabolic disease. Notably, even short-term dietary changes can rapidly reshape the microbiome, highlighting its adaptability. In contrast, diets high in fat and sugar, particularly those rich in fructose, promote dysbiosis, IR and hepatic fat accumulation. This intensifies inflammation and accelerates the development of NAFLD. Modern dietary patterns dominated by UPFS further exacerbate these effects by impairing intestinal barrier integrity and promoting chronic low-grade inflammation. Thus, diet, microbiome composition and metabolic health are tightly interconnected. Beneficial dietary patterns support microbial diversity and metabolic resilience, whereas westernised diets promote dysbiosis, inflammation and NAFLD progression.

## 4. NAFLD in Adolescents: Epidemiology, Vulnerabilities and Microbiome Links

### 4.1. NAFLD and Its Ongoing Relevance

**Figure 4:**
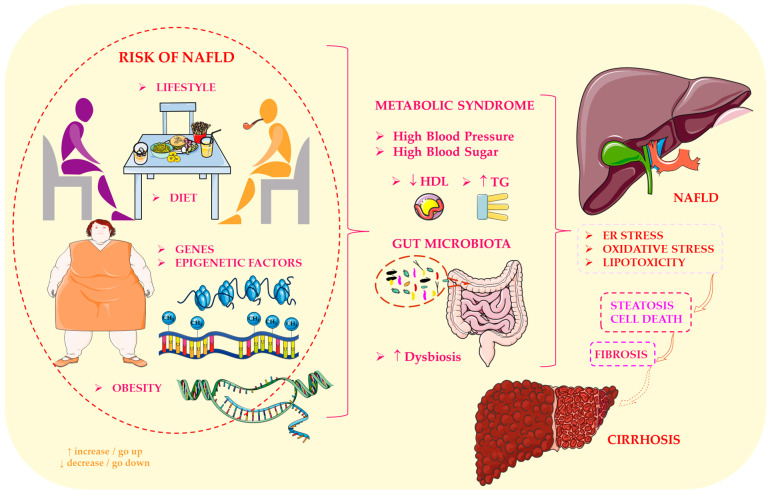
Multifactorial pathogenesis of non-alcoholic fatty liver disease (NAFLD). NAFLD develops as a result of complex interactions between diet, gut microbiota, and host metabolic and genetic susceptibility. Excess energy intake, obesity, and unhealthy dietary patterns promote gut dysbiosis, inflammation, oxidative stress, and lipid metabolism disturbances, leading to hepatic fat accumulation. These processes form a self-perpetuating cycle of metabolic dysfunction and chronic inflammation that drives disease progression from simple steatosis to non-alcoholic steatohepatitis (NASH), fibrosis, and ultimately cirrhosis. Image provided by Servier Medical Art (https://smart.servier.com/), licensed under CC BY 4.0 (https://creativecommons.org/licenses/by/4.0/, accessed on 10 January 2026). Abbreviations: HDL–high-density lipoprotein; NAFLD–non-alcoholic fatty liver disease; TG–triglycerides.

NAFLD has emerged as one of the most prevalent chronic liver disorders worldwide, affecting not only adults, but also an increasing number of children and adolescents. Essentially, NAFLD involves an abnormal accumulation of lipids within hepatocytes in individuals who do not consume alcohol at levels known to cause liver damage. While the condition may initially appear benign, it can progress to NASH, fibrosis, cirrhosis and hepatocellular carcinoma. This broad continuum highlights that NAFLD is a complex, multisystem disease with profound long-term consequences, not merely a metabolic curiosity. As demonstrated in Figure 4, the development of NAFLD is influenced by a variety of factors, including metabolic, dietary, genetic and microbiome-related elements.

The growing interest in NAFLD research is driven by several converging trends. Firstly, the global increase in obesity, IR and sedentary lifestyles has led to a dramatic rise in the prevalence of NAFLD, establishing it as one of the leading causes of chronic liver disease in young people. Secondly, NAFLD is closely associated with extrahepatic complications, including type 2 diabetes, cardiovascular disease and chronic kidney disease, highlighting its systemic nature. Thirdly, despite its high prevalence, NAFLD often remains clinically silent for years, resulting in a delayed diagnosis until irreversible liver damage has occurred. This silent progression underscores the importance of early detection and mechanistic understanding.

Recent studies have also revealed that NAFLD is not a uniform disease, but rather a heterogeneous condition influenced by genetic predisposition, diet, lifestyle and, crucially, the gut microbiota [72]. Recognising that intestinal dysbiosis can influence hepatic inflammation, lipid metabolism, and immune responses has opened new avenues for therapeutic intervention [73]. Consequently, NAFLD has emerged as a model disorder for investigating the complex interplay between host metabolism, microbial ecology, and environmental exposures.

These interactions are further modulated by dietary patterns. Individuals with NAFLD frequently consume diets that are high in saturated fats and low in essential micronutrients [73,74]. Both human and animal studies have shown that high-fat diets induce profound alterations in the gut virome, including increased activity of lytic phages, which exacerbates existing dysbiosis [75]. This phage-driven destabilisation of microbial communities may act synergistically with diet-induced metabolic stress, thereby intensifying inflammation and accelerating hepatic steatosis. In this context, the interplay between diet, bacteriophages and bacterial communities emerges as a critical determinant of NAFLD pathophysiology.

Thus, NAFLD is a rapidly evolving global health challenge that extends far beyond the liver. Its increasing prevalence among adolescents, stealthy progression and multifactorial nature make it a key area of focus for biomedical research. Continued investigation, particularly into the molecular mechanisms linking metabolism, immunity and the gut microbiome, offers the best opportunity to develop effective diagnostic tools and targeted therapies. In this sense, NAFLD is not only a disease of our time, but also a critical lens through which we can better understand the metabolic and microbial forces that shape human health.

A comparative analysis of children with NAFLD and obese peers without NAFLD was conducted by Schwimmer et al. [76]. This analysis used both clinical assessments and liver histology. They found that patients with NASH had higher levels of *Lactobacillus* and *Oribacterium*, while children with NAFLD without NASH had higher levels of *Oscillibacter*, *Lactobacillus*, *Akkermansia* and *Enterococcus* [76]. These results suggest that distinct microbial signatures may differentiate early-stage NAFLD from its progressive inflammatory form, indicating that specific taxa could contribute to-or reflect-the transition from simple steatosis to NASH in the paediatric population.

A broad body of evidence supports the association between gut microbiome composition and metabolic disorders such as obesity, IR, metabolic syndrome, type 2 diabetes, and NAFLD [77]. Comparative analyses across studies consistently show that these conditions are accompanied by dysbiosis, characterised by reduced microbial diversity and shifts towards pro-inflammatory or metabolically unfavourable taxa. For instance, scientific team [76] identified microbial patterns specifically linked to paediatric NAFLD phenotypes, whereas Festi et al. [77] emphasised the broader metabolic consequences of microbiome alterations, highlighting the systemic nature of gut–liver interactions.

It is known that microbiome disturbances are closely tied to impaired energy, lipid and glucose metabolism, as well as chronic, low-grade inflammation—factors that collectively promote the development and progression of metabolic diseases [78]. Together with the findings of Schwimmer et al. [76], these mechanistic insights suggest that the gut microbiota may influence hepatic pathology in adolescents via inflammatory pathways, including endotoxin-mediated immune activation, as well as through metabolic dysregulation. Furthermore, recent research indicates that the gut microbiome profile, encompassing taxonomic composition, functional gene content and microbially derived metabolites, represents a promising source of diagnostic and prognostic biomarkers for metabolic diseases, including NAFLD [72]. This aligns with earlier observations that specific microbial taxa differentiate NAFLD subtypes in young people, reinforcing the potential of microbiome-based tools for early detection and risk stratification. Importantly, integrating microbial biomarkers with clinical and biochemical parameters could improve the accuracy of NAFLD assessment in adolescents, for whom non-invasive diagnostic options are limited.

Thus, these studies converge on the central conclusion that alterations in the gut microbiome are intricately linked to metabolic dysfunction and liver pathology in young people. Therefore, understanding these microbial signatures may be pivotal in identifying early disease trajectories and developing targeted interventions aimed at restoring gut–liver homeostasis.

### 4.2. Microbiome Alterations Associated with NAFLD

The gastrointestinal tract is home to a vast and highly interactive microbial ecosystem that coexists symbiotically with its human host. This microbiota exerts a profound effect on human physiology, influencing health and disease. The colonic microbiota alone comprises a remarkably diverse community, with over one thousand bacterial species having been identified to date, although many remain poorly characterised. Approximately 160 species are typically present in each individual [79].

There is growing evidence that the gut microbiome plays a pivotal role in the pathogenesis and progression of paediatric NAFLD, largely through mechanisms involving oxidative stress, immune-neuroendocrine signalling, and impaired gut-liver axis homeostasis. Recent integrative analyses have revealed that dysbiosis in adolescents amplifies hepatic oxidative injury and disrupts systemic redox balance, thereby exacerbating the metabolic inflammation that is characteristic of NAFLD. Kurhaluk and colleagues have repeatedly demonstrated that gut microbial communities are central regulators of oxidative stress responses in various physiological contexts [80]. Their research into environmental stressors shows that the modulation of redox pathways by the microbiota determines host susceptibility to oxidative damage, emphasising the role of the microbiome as a dynamic buffer against exogenous insults [80]. Complementary findings on male reproductive physiology further emphasise that antioxidant–microbiota interactions shape systemic metabolic resilience, demonstrating how disturbances in microbial composition can propagate oxidative imbalance beyond the primary affected organ [81,82]. These mechanistic insights align with broader concepts emerging from neuroimmune research, in which the vagus nerve and parasympathetic signalling act as critical conduits linking gut microbial metabolites with central stress-regulatory circuits [83]. Such neuroimmune–microbial crosstalk is increasingly recognised as being relevant for adolescents with NAFLD, who frequently exhibit heightened stress reactivity and altered autonomic tone. Furthermore, the authors’ comprehensive review of the modulation of the microbiota–gut–brain axis by antioxidants provides a conceptual framework for understanding how targeted antioxidant strategies may restore microbial equilibrium, reduce oxidative stress and improve metabolic outcomes in young people with fatty liver disease.

In healthy individuals, the gut microbiota is dominated by the phyla *Bacteroidetes* (Gram-negative) and *Firmicutes* (Gram-positive), which together account for nearly 90% of all bacteria, while *Actinobacteria* constitute the remaining fraction [84]. However, despite this apparent stability, commensal bacteria can shift towards a parasitic or pathogenic phenotype under specific environmental or host-related conditions [85,86]. The composition, diversity and metabolic activity of the microbiota are dynamic features that collectively influence the onset, progression and responsiveness to treatment of numerous disorders [87].

Dietary patterns, particularly fibre, fat and protein intake, as well as the proportion of plant-based versus high-fat foods consumed, strongly influence the microbiome [88]. Early-life and environmental factors, including mode of delivery, infant feeding practices, antibiotic exposure, sleep patterns, stress and lifestyle, also shape microbial development. Furthermore, host-specific genetic and immunological traits modulate the structure and function of the microbiome [89].

Dysbiosis is typically characterised by reduced microbial diversity and altered proportions of dominant bacterial phyla. For example, some studies have reported an increased *Firmicutes*-to-*Bacteroidetes* ratio [63]. Two hallmark features of dysbiosis are frequently described. Firstly, there is a loss or marked reduction in beneficial commensals, which decreases microbial diversity and is associated with metabolic and immune-mediated disorders [90]. Secondly, there is overgrowth of potentially pathogenic commensals, or pathobionts. In healthy individuals, pathobionts constitute only a minor fraction of the microbiota. However, in many diseases, including NAFLD, Gram-negative *Enterobacteriaceae* (a subgroup of *Proteobacteria*) expand disproportionately. The proliferation of *Proteobacteria* is widely regarded as a diagnostic marker of dysbiosis and increased disease risk [63].

Disruption to the native gut microbiota can be caused by external factors such as HFD, sedentary behaviour and antibiotic use [91,92]. Under normal conditions, the microbiota exhibits resilience, preventing long-term dysbiosis through high microbial diversity and functional redundancy, which suppress the expansion of opportunistic microbes collectively. However, this resilience diminishes when disturbances occur during critical developmental periods, such as adolescence [93,94]. Persistent dysbiotic shifts are increasingly recognised as contributing to the pathogenesis of human disease [91,92].

Several studies have demonstrated that adolescents and other groups with NAFLD harbour distinct microbial signatures compared with healthy controls [95]. Altered abundances of *Bifidobacterium*, *Prevotella* and *Lactobacillus* have been reported; these changes may compromise intestinal barrier integrity and promote the translocation of endotoxins, such as LPS, thereby triggering hepatic inflammation [23,25]. It was shown that patients with NAFLD frequently present with a gut microbiota dominated by members of the phylum *Firmicutes*, resulting in an elevated *Firmicutes*-to-*Bacteroidetes* ratio compared with healthy controls [10,23,25]. These studies have highlighted that such compositional shifts are not merely descriptive markers, but may also reflect deeper metabolic disturbances, including altered SCFA production, impaired bile acid metabolism and enhanced energy harvest from the diet [96]. These findings reinforce the notion that NAFLD dysbiosis is characterised by both taxonomic imbalance and functional disruption.

Within this altered microbial ecosystem, bacteriophages—constituting the dominant component of the gut virome—play a central regulatory role by infecting and modulating bacterial populations [97]. During the lytic cycle, bacteriophages actively lyse bacterial cells, thereby limiting the overgrowth of specific taxa and contributing to microbial equilibrium [98]. Conversely, during the lysogenic cycle, bacteriophages integrate into the bacterial genome and remain dormant until reactivated by environmental triggers, such as an unhealthy diet or psychological stress [99]. As demonstrated in experimental and clinical studies, this can lead to widespread lysis of bacteria and the release of LPS, amplifying intestinal and systemic inflammation and ultimately promoting NAFLD progression [95,100]. These mechanistic insights highlight the significance of the gut virome as a frequently overlooked yet biologically important contributor to the gut–liver axis.

Altogether, the evidence suggests that NAFLD-associated dysbiosis cannot be fully understood without considering the dynamic interactions between bacteria, bacteriophages and dietary exposures. These interconnected factors shape the inflammatory and metabolic environment of the gut–liver axis, offering potential targets for future diagnostic and therapeutic strategies.

### 4.3. Microbiome Dynamics in Adolescence

Research has shown that the shift from childhood to adolescence is driven by the reactivation of neuroendocrine axes and the significant hormonal changes that are characteristic of puberty [94,101,102]. These endocrine shifts, particularly the rise in estrogen and testosterone, have been shown in clinical studies to correlate with marked differences in the composition and diversity of the gut microbiome [103,104]. Furthermore, mounting evidence suggests that the capacity of gut bacterial communities to metabolise or modify sex hormones varies, indicating that microbial activity may directly influence host physiology during this critical developmental period [103,104]. Together, these findings support a bidirectional relationship between sex hormones and the gut microbiota, with potential implications for healthy growth and pubertal maturation [94].

Recent clinical studies further demonstrate that the gut microbiota continues to mature towards an adult-like profile throughout adolescence, a process that is closely linked to pubertal hormonal dynamics [105,106]. While this developmental plasticity is essential for physiological maturation, it also renders the adolescent microbiome particularly vulnerable to disruption. Long-lasting or recurrent alterations in gut microbial communities during sensitive developmental periods may impair the ability of the microbiota to return to a homeostatic state with the host as shown in studies [91,92]. Consequently, exogenous disturbances, such as dietary changes, antibiotic exposure or environmental stressors, may induce persistent dysbiosis during adolescence with potentially detrimental long-term effects on metabolic and endocrine health [94].

One of the first research projects to describe the characteristics of the intestinal microbiota across the various stages of puberty stated that there were different microbial signatures linked to the pre-pubertal and pubertal stages are studies of team Yuan et al. [105]. It was shown that pre-pubertal individuals exhibited higher abundances of bacteria from the order *Clostridiales*, the family *Clostridiaceae*, and the genus *Coprobacillus*, whereas adolescents showed increased representation of taxa from the class *Betaproteobacteria* and the order *Burkholderiales*. Notably, these differences were independent of BMI-Z, indicating that pubertal stage itself, rather than adiposity, was the primary driver of microbial variation. Within the pubertal group, the abundance of *Adlercreutzia*, *Dorea*, *Ruminococcus*, *Clostridium* and *Parabacteroides* positively correlated with testosterone levels [105]. These associations suggest that sex hormones may selectively shape microbial communities, potentially influencing metabolic and endocrine pathways during adolescence.

The protective role of indole and its derivatives is highlighted by both Devlin et al. [48] and Beaumont et al. [49], who also demonstrate how these compounds strengthen epithelial tight junctions, reduce inflammation and improve glucose homeostasis. Whitfield-Cargile et al. [50] and Chimerel et al. [51] demonstrate that indole increases GLP-1 secretion, thereby enhancing metabolic regulation. Alexeev et al. [52] demonstrate that indole-3-aldehyde (IAA) activates AhR, inducing IL-22 and IL-10R1. Meanwhile, Natividad et al. [53] and Krishnan et al. [54] show that IAA suppresses pro-inflammatory cytokines and lipogenesis. Venkatesh et al. [55] and Abildgaard et al. [56] report that indole-3-propionic acid enhances barrier integrity and improves glucose metabolism.

The scientific team of Fennema et al. [57] describe how TMA, produced from dietary choline, betaine and carnitine, is converted to TMAO by hepatic flavin FMO enzymes. Barrea et al. [58] emphasise that TMA production is strongly influenced by dietary patterns commonly observed in obesity. Zhu et al. [59] demonstrate that TMAO regulates bile acid metabolism via CYP7A1 and hepatic transporters. Ji et al. [29] add that choline deficiency impairs VLDL secretion, thereby promoting triglyceride accumulation. Meanwhile, Warrier et al. [60] and Shih et al. [61] demonstrate that TMAO promotes IR, dyslipidaemia, and hepatic inflammation. Conversely, Oellgaard et al. [62] link TMAO to altered cholesterol homeostasis. The way in which SCFA and microbiota-driven bile acid modifications modulate FXR and TGR5 signalling, shaping lipid and glucose metabolism, was emphasised by Zhang et al. in 2024 [63].

Some authors, such as Aron-Wisnewsky et al. [25], also report a decrease in beneficial bacteria, including *Akkermansia muciniphila*, *Faecalibacterium prausnitzii* and *Bifidobacterium*. The intestinal barrier is strengthened by *A. muciniphila* via Amuc-1100-TLR2 signalling, as demonstrated by Plovier et al. [107]. Kimura et al. [108] report that SCFAs produced by this bacterium activate glucose-regulated protein (GRP) receptors, thereby improving energy metabolism. Li et al. [109] demonstrate that *F. prausnitzii* produces the anti-inflammatory compound butyrate, while *Bifidobacterium* enhances barrier integrity and promotes Treg-mediated immune tolerance.

In summary, adolescent NAFLD is characterised by a loss of beneficial taxa, an expansion of pro-inflammatory bacteria, and profound metabolic reprogramming. Dysbiosis amplifies hepatic stress through endotoxemia, altered SCFA and bile acid signalling, increased lipogenesis, and chronic inflammation, thereby accelerating progression towards NASH.

It is not yet fully clear what the practical consequences of these microbial differences are, but the observed links show that the gut microbiome may have an important role in controlling hormonal signals, metabolism, and how puberty develops. Further research is needed to clarify whether these microbial patterns contribute causally to metabolic outcomes or represent adaptive responses to hormonal changes. Nevertheless, the current evidence emphasises that adolescence is a critical period in which microbiome–hormone interactions may have long-lasting health consequences.

### 4.4. Microbial Dysbiosis and Metabolic Signatures of NAFLD Progression in Adolescents

It has long been known that the gut microbiota constitutes a complex microbial ecosystem that is essential for human health, influencing metabolic pathways, immune responses and the development of chronic diseases. Its composition is shaped by diet, lifestyle, genetics and exposure to medication, and disturbances to this balance—referred to as dysbiosis—have been widely reported to lead to reduced microbial diversity and the expansion of potentially pathogenic species. Such alterations are increasingly recognised as a key contributor to metabolic disorders, particularly NAFLD. Previous studies have shown that adolescents with NAFLD have markedly different levels of genera such as *Bifidobacterium*, *Prevotella*, and *Lactobacillus* compared to healthy peers, suggesting that early microbial shifts may influence disease susceptibility [105].

Previous studies have suggested that puberty is a critical period during which the gut microbiota continues to mature under the influence of sex hormones. In an Egyptian adolescent cohort, individuals with NAFLD exhibited higher abundances (above the 50th percentile) of *Bacteroides fragilis*, *Bifidobacterium* spp., *Escherichia coli*, *Lactobacillus* spp. and *Prevotella* spp. compared with healthy controls. Logistic regression analysis identified the presence of *Clostridium difficile* and *Salmonella* spp., as well as elevated levels of *Bifidobacterium* and *Prevotella* and reduced levels of *Lactobacillus*, as variables associated with NAFLD [110]. These findings highlight the heterogeneity of dysbiosis patterns across populations and suggest that both traditionally beneficial and pathogenic taxa may contribute to disease under specific ecological contexts. It is now understood that these hormonal changes can reshape microbial communities in ways that affect metabolic and inflammatory pathways, thereby altering the risk of liver disease [103,104]. Meanwhile, bacteriophages—the dominant viral entities in the gut—play a pivotal regulatory role. Some phages have been shown to promote microbial stability, while others contribute to inflammatory states that may accelerate NAFLD progression.

A metagenomic analysis of the gut virome in NAFLD patients has provided further insight into these interactions. As Lang et al. [111] reported, individuals with advanced hepatic fibrosis exhibited a marked reduction in phages infecting *Lactococcus* and *Leuconostoc*, a pattern that coincided with an increased abundance of these bacterial hosts. Conversely, phages targeting *Streptococcus*, *Escherichia*, *Enterobacteriaceae* and *Lactobacillus* were more prevalent, while their bacterial hosts’ populations declined. These reciprocal trends highlight what is becoming increasingly evident: the gut virome exerts a profound influence on the structure of the bacterial community during the progression of liver disease. There is growing evidence that adolescents with NAFLD have different gut microbiota compositions to healthy adolescents [5]. Notably, the adolescent microbiome differs from those of children and adults, suggesting that gut microbial communities mature during puberty, gradually acquiring an adult-like profile [94]. Liver diseases, including NAFLD, are consistently associated with shifts in microbial composition and function—collectively termed dysbiosis [112].

Earlier work by Michail et al. [113] involved comparing children with NAFLD and obese children with normal alanine aspartate aminotransferase (ALT) levels with non-obese controls. Children with NAFLD exhibited higher faecal ethanol concentrations and increased *Prevotella* abundance compared to both of the other groups. This aligns with more recent observations linking *Prevotella*-rich microbiomes to enhanced fermentation capacity and elevated production of metabolites that may exacerbate hepatic steatosis.

It has also been demonstrated that *Lactococcus* and *Leuconostoc*, microorganisms commonly found in fermented foods, may mitigate the severity of NAFLD by modulating the composition of the gut microbiota, reducing circulating LPS, enhancing SCFA production, improving lipid metabolism and attenuating inflammation. Together, these mechanisms lead to reductions in body weight, hepatic steatosis and IR [114,115,116,117,118]. Conversely, there is consistent evidence that *Streptococcus*, *Escherichia* and *Enterobacteriaceae* are associated with the development and progression of hepatic steatosis [25,119,120,121].

These findings illustrate what is now widely acknowledged: both bacterial and viral components of the gut ecosystem shape the metabolic and inflammatory environment of NAFLD in young people. The interplay between diet, hormonal maturation, bacterial taxa and bacteriophages creates a dynamic, highly responsive system in which even subtle shifts can have long-lasting consequences for liver health. To summarise these insights and highlight the diversity of research approaches, Table 2 presents a summary of representative studies examining the adolescent gut microbiota.

## 5. Diet, Microbiome Modulation and Metabolic Consequences

### 5.1. Dietary Patterns and Gut Microbiome Modulation

It was known that dietary composition is one of the strongest determinants of gut microbiome structure and function, and therefore plant-based and Mediterranean dietary patterns-characterised by high intakes of dietary fibre, vegetables, fruits, legumes and unsaturated fatty acids-promote favourable microbial shifts [125]. These diets enhance the production of SCFAs and reduce circulating TMAO, which are metabolic changes associated with reduced cardiometabolic risk. Consistent with these findings, Soldán et al. [126] reported that such dietary patterns increase microbial diversity, which is a key indicator of metabolic resilience. Namely, Khavandegar et al. [127] demonstrated that higher abundances of *Faecalibacterium*, *Prevotella*, and *Bacteroides* are associated with improved glycaemic control, reduced adiposity, enhanced bowel regularity, lower inflammation, and a decreased risk of hospitalisation. These results reinforce the concept that the gut microbiota acts as a central regulator of metabolic and gastrointestinal health.

Dietary interventions can induce rapid microbial shifts, with high-fat or high-sugar diets altering the microbiome within days. Beneficial changes also occur quickly: Bourdeau-Julien et al. [128] observed reductions in harmful metabolites after only two days of a Mediterranean diet. Similarly, Rejeski et al. [129] reported rapid increases in microbial diversity following the adoption of a Mediterranean diet. However, many of these changes are reversible, with microbial profiles returning to baseline levels once previous eating habits are resumed [130]. As Zhang [131] emphasised, diet influences microbial composition and function, including metabolite production, intestinal barrier integrity, and immune regulation.

It was shown that non-digestible plant polysaccharides and dietary fibre, which cannot be degraded by host enzymes, are fermented by gut microbes into SCFA-primarily acetate, propionate and butyrate-and these metabolites support colonocyte energy supply, modulate host metabolism, regulate nutrient absorption and influence intestinal motility [79]. In contrast, the fermentation of proteins and fats can generate harmful metabolites that affect immune function and intestinal health [132].

Therefore, plant-based and Mediterranean diets promote beneficial microbial profiles, increase SCFA production and enhance microbial diversity, whereas high-fat and high-sugar diets rapidly induce dysbiosis. These microbial shifts have direct metabolic consequences and form the basis for understanding how diet contributes to metabolic diseases such as NAFLD.

As the gut microbiome responds rapidly to dietary changes, the next section will examine how diet-induced dysbiosis and metabolic disturbances contribute to the development and progression of NAFLD.

### 5.2. Diet-Induced Dysbiosis and Pathways Linking Diet to NAFLD

The physicochemical conditions of the gut, particularly luminal pH, strongly influence the dominance of specific bacterial groups and the metabolic fate of dietary substrates. A lower colonic pH (pH ~5.5) favours the growth of *Firmicutes* and increases butyrate production, while simultaneously suppressing the growth of *Bacteroides* and reducing propionate formation [133]. Under mildly acidic conditions, species utilising the CoA-transferase pathway increase acetate consumption and butyrate synthesis [134]. Conversely, iron deficiency or altered oxygen availability can inhibit SCFA production and shift fermentation towards lactate [135].

Human gut microbiota ferment indigestible carbohydrates into SCFAs, primarily acetate, propionate and butyrate, which are subsequently absorbed and utilised by the host. Propionate and butyrate, in particular, exert multiple beneficial physiological effects [136]. Amino acid-based fermentation also contributes to SCFA pools; aspartate, alanine, threonine and methionine are major propionate precursors, while butyrate is predominantly derived from glutamate, lysine, histidine, cysteine and serine [79]. SCFAs typically reach concentrations of 50–200 mM in the human colon, where they act as energy substrates and regulators of gene expression via specific receptors [137,138]. The microbiome also modulates host metabolic pathways related to energy homeostasis, glucose and lipid metabolism, and bile acid turnover [139]. Although less than 1% of gut microbes ferment proteins, this process yields both SCFAs and potentially harmful metabolites [132]. The balance of substrates reaching the colon—10–30% of dietary proteins and ~5% of lipids—shapes microbial composition and metabolic outputs.

Butyrate is synthesised via glycolysis-derived acetyl-CoA, which is then condensed to acetoacetyl-CoA before being reduced to butyryl-CoA. The final step proceeds via either the butyryl-CoA:acetate CoA-transferase pathway or the phosphotransbutyrylase-butyrate kinase pathway. Butyrate-producing bacteria are mainly found in the families *Ruminococcaceae* and *Lachnospiraceae*, but also in *Erysipelotrichaceae* and *Clostridiaceae*. However, many dominant *Firmicutes* (e.g., *Blautia* spp. and *Ruminococcus* spp.) do not produce butyrate [136]. Propionate is generated via the succinate pathway (*Bacteroidetes* and *Negativicutes*) or the propanediol pathway from deoxy sugars, such as rhamnose and fucose [136,140]. As SCFAs directly influence hepatic metabolism, alterations in microbial composition or nutrient availability can propagate effects downstream on liver function. This link is particularly relevant in the context of NAFLD.

### 5.3. Microbial Metabolites, SCFA Production and Epigenetic Regulation

Deficiencies in micronutrients, particularly folate and vitamin B_12_, are strongly associated with the progression of NAFLD and an increased susceptibility to hepatic injury. Imbalances in vitamins correlate with gut dysbiosis, hepatic lipotoxicity, immune dysfunction, chronic inflammation, oxidative stress, genetic instability and epigenetic alterations that are characteristic of NAFLD [141]. Folate and vitamin B_12_ act as essential methyl-group donors for the synthesis of S-adenosylmethionine (SAM), which is required for DNA methylation [142]. Low folate and B_12_ levels independently predict the severity of NASH as studies shown [143], and the dysregulation of methyl-group donors can promote NAFLD by altering DNA methylation [144].

In mice, diets deficient in methyl donors have been shown to cause hypomethylation of 164 hepatic genes involved in lipid and glucose metabolism, DNA repair, fibrogenesis and tissue remodelling [144]. Such diets also intensified inflammation via activation of the kynurenine pathway [145]. However, supplementation with methyl-group donors (folate, betaine, choline and vitamin B_12_) protected rodents from diet-induced steatosis by reversing the methylation changes in genes that regulate lipid metabolism. These genes include peroxisome proliferator-activated receptor-α (PPARα), acetyl-CoA acyltransferase and fatty acid synthase (FASN) [146,147,148]. Therefore, micronutrients and endocrine regulators, such as adiponectin, play a pivotal role in hepatic lipid metabolism and can exacerbate or mitigate NAFLD, depending on their circulating levels and receptor activity.

### 5.4. Polyphenols, Adiponectin Signalling and Hepatic Metabolism

Adiponectin, which is produced by white adipose tissue and is the most abundant adipokine, regulates lipid and glucose metabolism, as well as modulating inflammatory responses [149]. Circulating at concentrations of 2–30 μg/mL (~0.01% of plasma proteins), adiponectin exists in the form of globular isoforms, as well as a full-length form that assembles into trimers, hexamers and high-molecular-weight (HMW) complexes. The HMW isoform exhibits the strongest metabolic activity and serves as a marker of insulin sensitivity [149]. By activating the APPL1-AMPK pathway, adiponectin increases lipid oxidation, reduces inflammation and improves insulin responsiveness [150].

Adiponectin acts via its receptors, AdipoR1 and AdipoR2, which activate AMPK and PPARα, respectively. This promotes FAO and reduces oxidative stress by increasing antioxidant enzyme activity superoxide dismutase (SOD) [149,151]. Adiponectin lowers serum lipids, suppresses gluconeogenesis and increases HDL via the upregulation of ABCA1 and APOA-I [152,153,154]. Loss of PPAR-α in hepatocytes disrupts FAO and promotes NAFLD, highlighting the importance of this pathway [155].

A wide range of dietary polyphenols beneficially modulate adiponectin signalling and hepatic metabolism, which is of particular translational relevance given adiponectin’s central role in lipid oxidation, insulin sensitivity and hepatic inflammation. Berberine is a well-studied example of this effect, as it suppresses TNF-α, IL-6, IL-1β, COX-2, NOS and NO production, reduces lipogenesis and gluconeogenesis, and improves hepatic lipid accumulation [156,157,158]. In addition, berberine activates Nrf2-dependent antioxidant pathways and increases adiponectin, AdipoR1/R2 and AMPK activity [159,160], while also modulating gut microbiota composition [161]. Through its combined anti-inflammatory, metabolic and microbiota-directed activity, berberine addresses several mechanisms that contribute to the progression of MASLD.

Catechins (EGCG, EGC, ECG and EC) found in green tea have strong antioxidant and anti-inflammatory properties [149,162]. They activate AMPK, reduce ACC and SREBP-1C expression, and elevate adiponectin levels [163,164]. Their metabolic impact is particularly noteworthy because catechins effectively blunt hepatic lipogenesis, a mechanism that becomes dysregulated early in the development of steatosis. Chlorogenic acid improves glucose and lipid metabolism via PPAR-α activation and increases adiponectin signalling [165,166]. This mechanism is clinically significant because enhancing PPAR-α-driven fatty acid oxidation directly counteracts hepatic lipid overload, particularly in individuals with IR or a HFD.

Curcumin has been shown to reduce oxidative stress, activate AMPK, suppress SREBP-1C and NF-κB, and consistently increase adiponectin levels in both clinical and animal studies [149,167]. The relevance of curcumin lies in its ability to repeatedly demonstrate improvements in liver enzymes and inflammatory markers, making it a practical adjunct for long-term metabolic management. Resveratrol enhances the activity of AMPK, PPAR-α and PGC-1α, improves insulin sensitivity, reduces hepatic triglyceride (TAG) accumulation and increases adiponectin and its receptors [168,169]. Its unique contribution stems from its ability to stimulate mitochondrial biogenesis, addressing a fundamental deficit in NAFLD pathophysiology that few dietary compounds can effectively target.

Thus, SCFA-mediated microbial metabolism, micronutrient-dependent epigenetic regulation and adiponectin-centred endocrine signalling form a tightly interconnected network. Dietary polyphenols further reinforce these pathways, offering a multifaceted strategy to mitigate NAFLD and improve metabolic health in adolescents and adults alike.

## 6. Integrated Interventions for NAFLD Prevention in Adolescents

### 6.1. Dietary Strategies

NAFLD is strongly influenced by dietary composition, energy balance, and metabolic regulation. Current recommendations suggest that daily energy intake should consist of 45–65% carbohydrates, 20–35% fats and 10–35% protein in order to minimise long-term metabolic risk [170]. Major clinical societies, including the European Association for the Study of the Liver (EASL), the European Association for the Study of Diabetes (EASD), the European Association for the Study of Obesity (EASO), the American Association for the Study of Liver Diseases (AASLD), the European Society for Clinical Nutrition and Metabolism (ESPEN) and the Asian Pacific Association for the Study of the Liver (APASL), recommend a weight reduction of 7–10% through hypocaloric diets of –500 to –1000 kcal/day and physical activity. While these societies offer different specific guidance (for example, EASL-EASD-EASO and APASL recommend limiting processed foods and fructose, whereas AASLD and ESPEN do not), they all emphasise the importance of sustainable dietary patterns [171].

**Figure 5:**
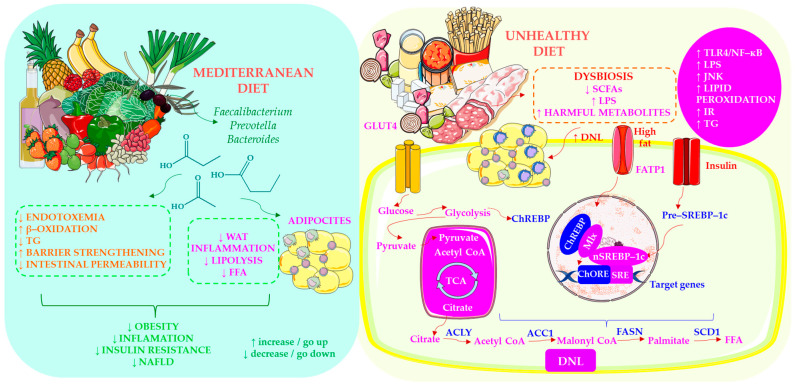
Contrasting effects of Mediterranean and ultra-processed diets on gut–liver axis and NAFLD development. The Mediterranean diet increases the abundance of SCFA-producing bacteria, strengthening intestinal barrier integrity, reducing endotoxemia and inflammation, and activating hepatic AMPK, which suppresses lipogenesis and enhances β-oxidation, thereby limiting triglyceride accumulation. In contrast, an ultra-processed diet induces dysbiosis, increases gut permeability, and promotes LPS-dependent TLR4/NF-κB activation, while high fructose intake further stimulates JNK, exacerbating insulin resistance, de novo lipogenesis, and lipid peroxidation. These processes increase FFA flux from WAT and drive hepatic steatosis progression. Image provided by Servier Medical Art (https://smart.servier.com/), licensed under CC BY 4.0 (https://creativecommons.org/licenses/by/4.0/, accessed on 10 January 2026). Abbreviations: ACC1–acetyl-CoA carboxylase 1; ACLY–ATP citrate lyase; ChORE–carbohydrate response element; ChREBP–carbohydrate-responsive element-binding protein; DNL–de novo lipogenesis; FASN–fatty acid synthase; FATP1–fatty acid transport protein 1; FFA–free fatty acids; GLUT4–glucose transporter type 4; IR–insulin receptor; JNK–c-Jun N-terminal kinase; LPS–lipopolysaccharide; Mlx–max-like protein X; NAFLD–non-alcoholic fatty liver disease; NF-κB–nuclear factor kappa B; nSREBP-1c–nuclear sterol regulatory element-binding protein-1c; Pre-SREBP-1c–precursor sterol regulatory element-binding protein-1c; SCFAs–short-chain fatty acids; SCD1–stearoyl-CoA desaturase 1; SRE–sterol regulatory element; TCA–tricarboxylic acid cycle; TG–triglycerides; TLR4–toll-like receptor 4; WAT–white adipose tissue.

The Mediterranean diet (MD) is the dietary pattern most consistently recommended for managing NAFLD [172]. The MD emphasises vegetables, fruits, whole grains, legumes, nuts, extra-virgin olive oil and fish, while limiting red meat and refined carbohydrates. Compared with low-fat or high-carbohydrate diets, the MD reduces hepatic steatosis and improves insulin sensitivity, even without weight loss [173]. In the PREDIMED randomised controlled trial (RCT), an MD supplemented with extra-virgin olive oil or nuts was found to reduce the incidence of hepatic steatosis and slow NAFLD progression [174,175]. As shown in Figure 5, Mediterranean and ultra-processed diets exert markedly different influences on the gut–liver axis, shaping NAFLD development through opposing metabolic and inflammatory pathways.

The MD provides monounsaturated fatty acids (MUFA), polyunsaturated fatty acids (PUFA) and polyphenols, which reduce DNL, enhance mitochondrial FAO and improve insulin signalling. Observational studies consistently demonstrate an inverse association between adherence to the MD and NAFLD severity [173], as well as a reduction in liver fat and stiffness [176,177,178,179,180,181,182,183,184]. The MD diet may also reduce the risk of hepatocellular carcinoma (HCC) and liver-related mortality [185,186].

The DIRECT-PLUS RCT demonstrated that a calorie-restricted MD combined with polyphenols (green tea and Mankai) produced greater reductions in liver fat than MD alone [183], highlighting the synergistic effects of plant bioactives. Thus, the practical and clinical relevance of MD has been demonstrated. It is feasible for adolescents, improves metabolic flexibility and reduces hepatic fat, even without weight loss, making it a powerful first-line intervention.

High-protein diets (HPDs) have shown promising effects on intrahepatic lipid content (IHLC). In a RCT, Markova et al. demonstrated that two isocaloric diets containing 30% protein—either animal-based or plant-based—reduced IHLC by 36–48% in individuals with type 2 diabetes [187]. Xu et al. [188] reported similar reductions with both high-protein (30%) and moderate-protein (20%) diets. Notably, plant-based proteins appear to be metabolically superior due to their lower saturated fat content and beneficial amino acid profiles [170,189].

Low-carbohydrate diets (LCD), including very-low-carbohydrate ketogenic diets (VLCD), reduce hepatic fat by lowering insulin secretion, suppressing DNL and increasing FAO. A VLCD reduced IHLC by 31% and improved insulin resistance by 57% in adults with NAFLD [190]. In adolescents, LCDs produced greater reductions in body weight, IHLC and IR than high-carbohydrate diets [191]. However, some LCD variants may increase low-density lipoprotein cholesterol (LDL-C) [192], necessitating careful monitoring.

Low-fat diets (LFD) can improve liver histology when a caloric deficit is maintained) [193]. However, high carbohydrate intake, especially of refined carbohydrates, may worsen hepatic fat accumulation [194]. The type of fat consumed is important: saturated and trans fats promote steatosis, whereas PUFAs reduce hepatic fat [195,196]. Therefore, both LCD and HPD can reduce liver fat, but long-term safety in adolescents requires the inclusion of plant-based protein sources and high-quality fats.

Hypocaloric diets remain the most effective strategy for reducing liver fat [197]. A daily deficit of 500–1000 kcal or an intake of ~1200 kcal for women and ~1400–1500 kcal for men improves body weight, liver enzymes, visceral fat and IHLC [171,198,199]. These benefits can persist for up to two years, even with partial weight regain [200]. Weight loss correlates strongly with histological improvement, including the resolution of NASH and the regression of fibrosis [192]. Caloric restriction is more effective than manipulating macronutrients alone [201].

Dietary fibre also plays a significant role. Insoluble fibre intake of at least 7.5 g/day improves fibrosis indices [202], while fruit-derived fibre intake of at least 8.8 g/day improves liver enzymes. Increasing fibre intake from 19 to 29 g/day reduces zonulin levels, improves liver enzymes and decreases steatosis, likely by strengthening intestinal barrier integrity [203].

Plant-based diets improve glycemic control and liver function in cases of diabetes, cirrhosis and hepatic encephalopathy [170]. Soy protein reduces ALT in NAFLD [204] and avoids the glomerular hyperfiltration associated with animal protein. Importantly, caloric deficit is the strongest predictor of NAFLD improvement. Fibre and plant-based proteins enhance metabolic outcomes and support gut–liver axis integrity.

### 6.2. Microbiome-Targeted Interventions

As the gut–liver axis plays a central role in NAFLD pathogenesis, microbiome-directed therapies, including probiotics, prebiotics, synbiotics and postbiotics, modify microbial composition, reduce endotoxemia and improve intestinal barrier function [24,91,205]. These interventions complement dietary strategies and can improve metabolic outcomes.

Probiotics from the species *Lactobacillus*, *Bifidobacterium*, *Streptococcus thermophilus* and *Saccharomyces* show hepatoprotective effects [206]. Multi-strain formulations preserve tight-junction proteins zonula occludens-1 (ZO-1) and ZO-2, and reduce hepatic triglycerides in HFD models [207]. *Lactobacillus casei Shirota* reduces LPS levels, inflammation, and steatosis via TLR4 inhibition and peroxisome proliferator-activated receptor gamma (PPARγ) activation [207]. *Lactobacillus rhamnosus* GG has been shown to suppress nuclear factor kappa-B (NF-κB), reduce inflammation and improve insulin sensitivity [208,209,210]. Other *Lactobacillus* species, including *L. acidophilus* L1, *L. paracasei*, *L. johnsonii* BS15, *L. reuteri* GMNL-263 and *L. gasseri* BNR17, also demonstrate benefits [207]. However, *Lactococcus lactis* subsp. *cremoris* may outperform LGG in reducing steatosis and inflammation [115].

A meta-analysis of 1555 patients with NAFLD found that probiotics improved BMI, ALT, AST, gamma-glutamyltransferase (GGT), insulin, homeostatic model assessment of insulin resistance (HOMA-IR) and total cholesterol, though effects on fasting glucose and TNF-α were inconsistent [206].

**Figure 6:**
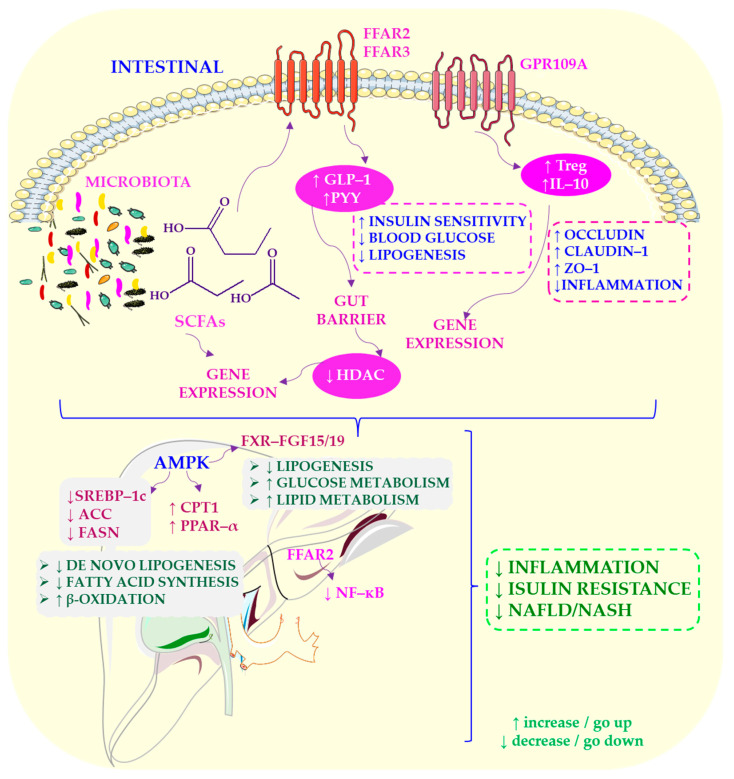
Protective effects of short-chain fatty acids against NAFLD through metabolic, immune, and epigenetic mechanisms. Short-chain fatty acids (SCFAs) protect against non-alcoholic fatty liver disease (NAFLD) by activating AMPK, suppressing hepatic lipogenesis, and attenuating inflammation through inhibition of NF-κB signaling. In parallel, SCFAs improve intestinal barrier integrity, modulate FXR-dependent bile acid signaling, and enhance GLP-1 secretion, thereby integrating metabolic, immunological, and epigenetic regulatory pathways within the gut–liver axis. Image provided by Servier Medical Art (https://smart.servier.com/), licensed under CC BY 4.0 (https://creativecommons.org/licenses/by/4.0/, accessed on 10 January 2026). Abbreviations: ACC–acetyl-CoA carboxylase; AMPK–AMP-activated protein kinase; CPT1–carnitine palmitoyltransferase 1; FASN–fatty acid synthase; FFAR2–free fatty acid receptor 2; FFAR3–free fatty acid receptor 3; FXR–FGF15/19–farnesoid X receptor–fibroblast growth factor 15/19 axis; GLP-1–glucagon-like peptide-1; GPR109A–G protein-coupled receptor 109A; HDAC–histone deacetylase; IL-10–interleukin-10; NAFLD–non-alcoholic fatty liver disease; NASH–non-alcoholic steatohepatitis; NF-κB–nuclear factor kappa B; PPAR-α–peroxisome proliferator-activated receptor alpha; PYY–peptide YY; SCFAs–short-chain fatty acids; SREBP-1c–sterol regulatory element-binding protein-1c; Treg–regulatory T cells; ZO-1–zonula occludens-1.

Prebiotics such as fructooligosaccharides (FOS) and inulin have been shown to reduce steatosis and inflammation in animal models [207,211]. They suppress lipogenic gene expression, increase SCFA production and improve microbiota composition [212,213]. Inulin reduces ALT and lipogenic gene expression while increasing butyrate production [214,215]. Figure 6 summarizes how SCFAs contribute to NAFLD protection by modulating metabolic pathways, immune responses and epigenetic regulation.

Synbiotics combine probiotics and prebiotics to enhance SCFA production. In animal models, synbiotics have been shown to reduce steatosis and insulin resistance [216,217]. Mechanistically, synbiotics activate PPARα-mediated FAO and suppress SREBP-1c and FAS-dependent lipogenesis [218]. The practical and clinical relevance of microbiome-targeted therapies is that they improve metabolic, inflammatory, and hepatic parameters, and they may be especially useful in adolescents with dysbiosis or poor dietary quality.

### 6.3. Lifestyle Approaches

Physical activity enhances the benefits of hypocaloric diets and reduces liver fat independently of weight loss [219,220]. Exercise improves peripheral insulin sensitivity, reduces DNL and decreases the flux of FFA to the liver, while increasing mitochondrial FAO. Studies have shown that both aerobic and resistance training reduce hepatic steatosis. Benefits are seen with 90–300 min of activity per week, and the guidelines recommend 150–300 min of moderate or 75–150 min of vigorous exercise [220]. Multiple studies have shown that exercise reduces IHLC and liver injury markers [221,222,223,224], with no significant differences observed between exercise types [225,226,227].

A valuable addition to this narrative is provided by the growing body of evidence linking the lifestyle-dependent activation of cytoprotective pathways, particularly Nrf2, to the metabolic disturbances observed in adolescents with NAFLD. Authors emphasise that antioxidant-rich functional foods, when combined with regular physical activity, have a synergistic effect on redox homeostasis, enhancing Nrf2-driven transcription of detoxifying and antioxidant enzymes [228]. Their analysis shows that dietary polyphenols, carotenoids and other bioactive compounds modulate not only gut microbial composition, but also potentiate mitochondrial resilience and lipid metabolism through Nrf2–Keap1 signalling. This is highly relevant for paediatric NAFLD, where impaired Nrf2 responsiveness and chronic low-grade oxidative stress are increasingly recognised as early mechanistic drivers of steatosis and hepatocellular vulnerability. Furthermore, this authors and others highlight that structured exercise amplifies these benefits by promoting metabolic flexibility, improving insulin sensitivity and stimulating myokine-mediated communication with the gut-liver axis. Integrating these insights with current microbiome-focused models suggests that targeted nutritional and physical activity interventions may restore redox balance, reshape dysbiotic microbial communities, and attenuate the progression of fatty liver disease in adolescents. In this context, the works provides a mechanistic bridge between lifestyle medicine, antioxidant biology and microbiota-dependent modulation of metabolic health. This reinforces the concept that Nrf2-centred strategies may be a promising addition to the management of NAFLD in young people [228,229,230].

It has been demonstrated that resistance training is particularly beneficial for unfit adolescents, as it provides comparable hepatic benefits with lower energy expenditure [231]. Exercise also improves VLDL clearance [232] and appetite regulation [171], as well as increasing muscle mass and counteracting sarcopenia, which is a risk factor for NAFLD and fibrosis [229,230]. The greatest reductions in body weight, visceral fat, and liver fat are achieved by combining MD with moderate exercise [219,233,234]. Studies emphasise that weight loss of ≥10% can almost resolve NASH and improve fibrosis, while a loss of even 5% can significantly improve NAFLD activity scores [22]. Lifestyle interventions reduce liver fat, waist circumference and LDL, achieving remission rates of ~50% with 3–5% weight loss and ~70% with 7–10% [219]. The practical and clinical relevance of exercise as a low-cost, accessible intervention that improves hepatic and metabolic health, even without weight loss, is critical for adolescents who may struggle with dietary adherence. Microbiome-directed therapies and physical activity complement dietary interventions by improving gut–liver axis function, reducing inflammation, and enhancing metabolic flexibility. Combining them offers a comprehensive, non-pharmacological strategy for NAFLD management in adolescents.

### 6.4. Equity and Adolescent Adherence Barriers

Equity-related factors also play a critical role in determining the effectiveness of lifestyle and microbiome-targeted interventions for adolescents [5,6,9]. Young people often face substantial barriers to adherence, including limited autonomy over food choices, socioeconomic constraints affecting access to healthy foods, inconsistent family support, time pressures related to school and fewer opportunities for structured physical activity. Psychosocial factors such as concerns about body image, stress, stigma and mental health issues may further hinder sustained engagement with dietary or activity-based interventions [11,12]. These equity-linked barriers demonstrate that evidence-based strategies may have limited real-world impact unless adapted to the developmental, social and environmental contexts in which adolescents live.

Recent studies indicate that adolescents exhibit increased sensitivity to rewarding stimuli, including alcohol, sugary and fatty foods, and psychoactive substances. This is due to heightened dopaminergic activity within the mesolimbic reward system, as shown in references [235,236,237]. This neurobiological vulnerability promotes risk-taking behaviors and contributes to the development of addictive patterns, which are strongly associated with obesity and metabolic disturbances. Alcohol consumption has been identified as an important factor in exacerbating hepatic steatosis in young people, as chronic intake disrupts lipid metabolism and impairs liver function, with mechanisms that partially overlap with those observed in drug abuse. Similarly, frequent consumption of energy-dense junk food increases the risk of obesity and may accelerate the onset of NAFLD. Thus, these shared metabolic and neurobehavioural pathways highlight the interplay between reward-driven behaviours, central nervous system function, and liver health in adolescents.

This review has several limitations. Much of the available evidence on dietary, microbiome-targeted and physical activity interventions in NAFLD comes from adult populations, making full extrapolation to adolescents difficult given their distinct metabolic, hormonal and microbiome profiles. Many paediatric studies rely on small sample sizes, short-term interventions or heterogeneous methodologies, and diagnostic approaches vary widely—from ultrasound to MRI-PDFF and biochemical surrogates—limiting cross-study comparability and long-term inference. A quantitative meta-analysis of microbiome signatures was not feasible due to substantial heterogeneity in sequencing platforms, taxonomic resolution and reporting standards. Many primary studies also fail to adequately control for key confounders, such as recent antibiotic exposure, pubertal stage, medication use and co-existing metabolic conditions. Insights into mechanistic pathways such as DNL, FAO, AMPK activation and LPS–TLR4–NF-κB signalling are largely derived from animal models and may not fully reflect adolescent physiology. Adolescent-specific interventional data remain scarce, with most trials involving small samples or short follow-up periods. As this is a narrative review, it does not follow PRISMA methodology and is subject to an inherent risk of selection bias, although major databases and consistent eligibility criteria were used to mitigate this. Finally, few studies address behavioural, psychosocial, or family-level factors that strongly influence diet quality, physical activity, and microbiome stability in young people.

Future research should prioritise longitudinal studies tracking microbiota–metabolism interactions across adolescence, in order to clarify the causal pathways underlying the development of NAFLD. Because most studies published before 2023 use the term NAFLD, this terminology is retained when referring to original research, but future investigations should align with the updated MASLD nomenclature to ensure consistency with current clinical definitions. Multi-omics approaches that integrate metagenomics, metabolomics, and lipidomics are required to better characterise functional microbial alterations and their metabolic consequences. Standardisation of microbiota sampling, sequencing and analytical methods would also improve comparability across studies. Furthermore, well-designed dietary and microbiota-targeted intervention trials, such as those involving prebiotics, probiotics, synbiotics or precision nutrition strategies, are essential to determine whether modifying gut microbial ecology can meaningfully alter disease trajectories. Finally, research should explore age-specific therapeutic windows and identify microbial or metabolic biomarkers that could guide early prevention in at-risk adolescents.

## 7. Conclusions

Early lifestyle intervention remains the cornerstone of preventing and managing NAFLD in adolescents. Current evidence emphasises the important roles of dietary quality, gut microbiota composition, and lipid metabolism in determining disease risk and progression. Improving dietary patterns, increasing fibre intake, promoting regular physical activity, and investigating microbiome-targeted strategies are all promising ways of reducing hepatic fat accumulation and improving metabolic health in young people. Although several nutritional and microbial approaches show potential, further research is needed to identify the most effective, age-appropriate interventions and to clarify the mechanisms linking diet, microbiota and liver metabolism. A personalised, integrative approach combining dietary modification, physical activity and microbiome modulation is likely to be the most beneficial for long-term prevention.

## Figures and Tables

**Table 1:** Systematic summary of experimental and clinical investigations linking gut microbiota alterations to lipid metabolism in youth with NAFLD (2015–2025).

Study Context and Model Specification	Experimental Approach (In Vitro/In Vivo)	Age Group/Experimental Model Parameters	Major Observations and Pathophysiological Relevance	References
Gut microbiota, glucose, lipid, and water electrolyte metabolism in children with NAFLD	in vivo (clinical observational)	75 children, 7–16 years	Gut dysbiosis, reduced bacterial diversity, correlations with lipid and glucose metabolism in NAFLD	[30]
Effect of gut microbiota and PNPLA3 rs738409 variant on NAFLD in obese youth	in vivo observational clinical study	73 obese young patients	Altered *Firmicutes*/*Bacteroidetes* ratio; microbiota associated with hepatic fat fraction (HFF)	[31]
Integrative metabolomics of gut microbiota metabolites in child NAFLD	in vitro + in vivo (metabolomics analysis)	Children with NAFLD	Identification of microbiota-derived metabolites linked to lipid metabolism; validation in hepatocyte cell lines	[32]
Gut microbiota and cytokines in pediatric NAFLD	Human observational cohort study	89 children and adolescents, 5–15 years	Alterations in SCFA-producing microbiota; associations with cytokines and lipid metabolism	[33]
Non-alcoholic fatty liver disease and the gut microbiota in adolescents	Human observational cohort study	100 adolescents, 14–18 years	Microbiota differences (↑ *Bifidobacterium*, *Prevotella*; ↓ *Lactobacillus*)	[5]
Gut microbiota profiling in pediatric NAFLD and obesity	Clinical observational	115 children and adolescents	Dysbiosis in NAFLD vs. controls; changes in *Oscillospira* and other genera	[34]
Gut microbiota in adolescents and hepatic fat fraction (EPOCH)	Clinical observational	107 adolescents	Lower α-diversity associated with higher HFF; microbiota linked to metabolic parameters	[35]
In vitro and in vivo models of NAFLD	Review of preclinical models	Human hepatic cell lines + animal models (mice, rats)	Cell-based and murine models useful for studying lipid metabolism and microbiota interactions	[36]
Integrated gut–liver on a chip platform (in vitro NAFLD model)	in vitro (GLA platform)	Organ-on-a-chip	Simulation of the gut–liver axis for studying NAFLD mechanisms, including lipid metabolism and microbiota	[37]

Abbreviations: NAFLD—Non-alcoholic fatty liver disease; SCFA—Short-chain fatty acids; HFF—Hepatic fat fraction; GLA—Gut–Liver Axis; PNPLA3—Patatin-like phospholipase domain-containing protein 3; ↑ indicates increase, ↓ indicates decrease.

**Table 2:** Changes to the gut microbiota of adolescents and other youth populations with non-alcoholic fatty liver disease (NAFLD).

Study Population and Age Characteristics	Analytical Approach to Gut Microbiota Profiling	Principal Outcomes and Microbiome-Related Mechanistic Insights	References
100 obese adolescents with NAFLD vs. 100 healthy controls (14–18 years)	Culture-based microbiota analysis	Differences in the presence of *Clostridium difficile* and *Salmonella* spp.; higher abundance of *Bifidobacterium*/*Prevotella* and lower *Lactobacillus* in NAFLD	[5]
Children with NAFLD/NASH vs. controls	16S rRNA sequencing	Reduced α-diversity in NAFLD; microbiome composition associated with inflammation severity and fibrosis	[76]
Children with NAFLD/NASH	16S rRNA sequencing and metabolite profiling	Lower abundance of butyrate-producing bacteria (*Faecalibacterium*, *Roseburia*, *Coprococcus*) in NASH; alterations linked to glucose and lipid metabolism	[30]
25 obese with NAFLD, 18 obese without NAFLD, 15 healthy (9–17 years)	Shotgun metagenomics	Increased abundance of Proteobacteria/Gammaproteobacteria in NAFLD; reduced *Alistipes* in obese NAFLD; functional pathway differences	[122]
61 NAFL, NASH, obese and 54 controls patients	16S rRNA + VOC metabolite profiling	Decreased *Oscillospira* and increased *Ruminococcus*, *Dorea* in NAFLD; VOC metabolite shifts discriminating patients from controls	[34]
Children with NAFL/NASH vs. obese and healthy controls	Metabolomics + microbiome analysis	318 metabolites altered in NAFLD; associations of *Butyricicoccus* and *Alistipes* with ALT/AST levels and inflammation	[32]
Pediatric NAFLD	Microbiome review	Dysbiosis associated with disease severity and potential inflammatory mechanisms	[123]
52 children with MAFLD vs. 52 healthy (5–11 years)	16S rRNA + PICRUSt	Reduced α-diversity in MAFLD; decreased *Akkermansia*, *Blautia*, *Coprococcus* compared with controls	[29]
Children with NAFLD vs. controls	16S rRNA sequencing	Dysbiosis with higher abundance of *Collinsella*, *Escherichia–Shigella* and reduced *Bacteroides*, *Akkermansia* in NAFLD	[124]
Obese children with NAFLD vs. healthy controls	16S rRNA + metagenomics/metabolomics	Enhanced pathways of energy metabolism and bacterial alcohol production in NAFLD; altered SCFA profiles; increased hepatic fat content	[113]

Abbreviations: NAFLD—Non-alcoholic fatty liver disease; NASH—Non-alcoholic steatohepatitis; NAFL—Non-alcoholic fatty liver; MAFLD—Metabolic dysfunction-associated fatty liver disease; ALT—Alanine aminotransferase; AST—Aspartate aminotransferase; SCFA—Short-chain fatty acids; VOC—Volatile organic compounds; PICRUSt—Phylogenetic Investigation of Communities by Reconstruction of Unobserved States.

## Data Availability

No new data were created or analyzed in this study.
